# HuH-7-Lunet BLR Cells Propagate Rat Hepatitis E Virus (HEV) in a Cell Culture System Optimized for HEV

**DOI:** 10.3390/v14051116

**Published:** 2022-05-23

**Authors:** Mathias Schemmerer, Monika Erl, Jürgen J. Wenzel

**Affiliations:** National Consultant Laboratory for HAV and HEV, Institute of Clinical Microbiology and Hygiene, University Medical Center Regensburg, 93053 Regensburg, Germany; monika.erl@ukr.de (M.E.); juergen.wenzel@ukr.de (J.J.W.)

**Keywords:** rat HEV, *Orthohepevirus C*, hepatitis E virus, zoonosis, cell culture

## Abstract

The family *Hepeviridae* comprises the species *Orthohepevirus A–D* (HEV-A to -D). HEV-C genotype 1 (HEV-C1, rat HEV) is able to infect humans. This study investigated whether an optimized HEV-A cell culture system is able to propagate the cell culture-derived rat HEV, and if de novo isolation of the virus from rat liver is possible. We tested the liver carcinoma cell lines PLC/PRF/5, HuH-7, and HuH-7-Lunet BLR for their susceptibility to HEV-C1 strains. Cells were infected with the cell culture-derived HEV-C1 strain R63 and rat liver-derived strain R68. Cells were maintained in MEMM medium, which was refreshed every 3–4 days. The viral load of HEV-C1 was determined by RT-qPCR in the supernatant and expressed as genome copies per mL (c/mL). Rat HEV replication was most efficient in the newly introduced HuH-7-Lunet BLR cell line. Even if the rat HEV isolate had been pre-adapted to PLC/PRF/5 by multiple passages, replication in HuH-7-Lunet BLR was still at least equally effective. Only HuH-7-Lunet BLR cells were susceptible to the isolation of HEV-C1 from the liver homogenate. These results suggest HuH-7-Lunet BLR as the most permissive cell line for rat HEV. Our HEV-C1 cell culture system may be useful for basic research, the animal-free generation of large amounts of the virus as well as for the testing of antiviral compounds and drugs.

## 1. Introduction

The genus *Orthohepevirus* in the family *Hepeviridae* comprises the four species *Orthohepevirus A–D*. While birds and bats are the only known hosts for *Orthohepevirus B* and *D*, respectively, *Orthohepevirus A* and *C* infect a broader spectrum of mammals [1]. *Orthohepevirus A*, also known as hepatitis E virus (HEV), is by far the dominant species globally with 939 million individuals having experienced HEV infection and 15–110 million with recent or ongoing infection [2]. Lately, it has become evident that *Orthohepevirus C* (HEV-C) not only infects rats [3] and ferrets [4], but also has the zoonotic potential to infect humans [5]. *Orthohepevirus C* is currently classified into two genotypes (HEV-C1 and C2) and two further genotypes have been proposed (HEV-pC3 and HEV-pC4) [6]. HEV-C1 is also known as rat HEV and so far, human infections have only been described with this genotype. HEV-C2 is found in ferrets, while HEV-pC3 infects murids (Chevrier’s field mouse, *Apodemus chevrieri*) and HEV-pC4 infects cricetids (Père David’s vole, *Eothenomys melanogaster*) [7]. Moreover, a variety of other, unclassified HEV-C are also found in diverse genera of *Cricetidae* [6]. The first human infection with rat HEV was reported in 2018 by Sridhar et al. in Hong Kong [5], which was shortly followed by a second case of a Canadian patient who contracted the infection in Central Africa [8]. Lately, seven more human cases were found in Hong Kong [9] and a surveillance of HEV-C1 identified eight further infections, raising the total number to 16 in this city [10]. The first cases in Europe were reported from Spain only recently [11]. In Germany, despite some preliminary serological evidence from a study on forestry workers [12], no acute PCR-positive individuals have been identified thus far. In particular, a recent study on left-over samples from patients with suspected acute hepatitis E and no detectable HEV RNA found no evidence for infections with rat HEV [13].

Initial rat HEV isolation attempts yielded no viral growth in three different rat liver cell lines [14]. Subsequent efforts led to the successful propagation in the human hepatoma cell lines PLC/PRF/5, HuH-7, HepG2, HepG2/C3A, and human colorectal adenocarcinoma cell line Caco-2, while the human lung cancer cell line A549 only supported the viral growth of a strain derived from human feces [5,15,16]. Furthermore, a reverse genetic system of the HEV-C1 strain R63/DEU/2009 was established and replicated in PLC/PRF/5 [17]. However, recent studies still rely on virus stock generation in animals [18].

We asked whether our PLC/PRF/5-based cell culture system optimized for HEV propagation [19]—compared to HuH-7 and HuH-7-Lunet BLR—was (i) able to propagate the cell culture-derived rat HEV, and (ii) whether it was susceptible to de novo isolation of the virus from rat liver tissue.

## 2. Materials and Methods

### 2.1. Cell Culture

Liver carcinoma cell lines PLC/PRF/5 (ATCC CRL-8024), HuH-7, and subclone HuH-7-Lunet BLR (both kindly provided by Prof. Dr. Ralf Bartenschlager) [20] were maintained in BMEM (Eagle minimum essential medium (MEM) supplemented with 10% heat-inactivated fetal calf serum, 2 mM l-glutamine, 1% non-essential amino acids, 100 U/mL penicillin G and 100 µg/mL streptomycin; all reagents purchased from PAN Biotech (Aidenbach, Germany)). HuH-7-Lunet BLR cells were selected HuH-7 cells that were transfected with the hepatitis C virus (HCV) and supported replication. These cells were cleared from HCV to generate a subclone that is highly permissive to HCV replication. The HEV-C1 positive cells were expanded from T12.5 to T75 flasks, followed by another expansion step in T175 flasks and maintained until the pre-expansion viral load was detected by RT-qPCR in the supernatant or to a maximum of 10 weeks after the first expansion. Cells were then frozen and aliquots stored in liquid nitrogen.

### 2.2. Viruses and Inoculant

HEV-C1 strain R63 passages 1 [17] and 6 (passaged in PLC/PRF/5) and R68-positive liver homogenate [21] were kindly provided by Prof. Dr. Reimar Johne. Samples were diluted with PBS without Ca^2+^/Mg^2+^ containing 0.2% BSA (Sigma-Aldrich, St. Louis, MO, USA) (*w*/*v*), which also served as a negative control. R63 passage 2 was derived from the first isolation attempt in this study.

### 2.3. Viruses Passaging and Isolation

Virus inoculation was performed according to the protocol of our cell culture system optimized for HEV [19]. Briefly, cells were seeded in T12.5 flasks at a concentration of 1.0 × 10^5^ viable cells per cm^2^ and incubated at 37 °C and 5% CO_2_. Cells were maintained in MEMM (BMEM additionally supplemented with 2.5 µg/mL amphotericin B and 30 mM MgCl_2_) and the medium was refreshed every 3–4 days. Two weeks later, overconfluent cells were inoculated with 250 µL of HEV-C1-positive material and the negative control for 75 min at room temperature. Afterward, 2.5 mL of MEMM was added and the cells were incubated at 34.5 °C and 5% CO_2_. Twenty-four hours later, the supernatant was completely replaced with fresh MEMM and from then on, every 3–4 days.

### 2.4. HEV-C1 Quantification

The cell culture supernatants were collected from each flask weekly and at days 1 and 4 post inoculation. Nucleic acid was isolated on an EZ1^®^ Advanced XL workstation using the EZ1 Virus Mini Kit v2.0 (Qiagen, Hilden, Germany). Eluted nucleic acid was analyzed by RT-qPCR according to a published protocol [13]. The HEV-C1 RNA concentration was determined by absolute quantification and expressed as genome copies per mL (c/mL). The 95% limit of detection (LoD 95) was determined previously by probit analysis at 6.73 c/reaction (808 c/mL).

## 3. Results

**Figure 1:**
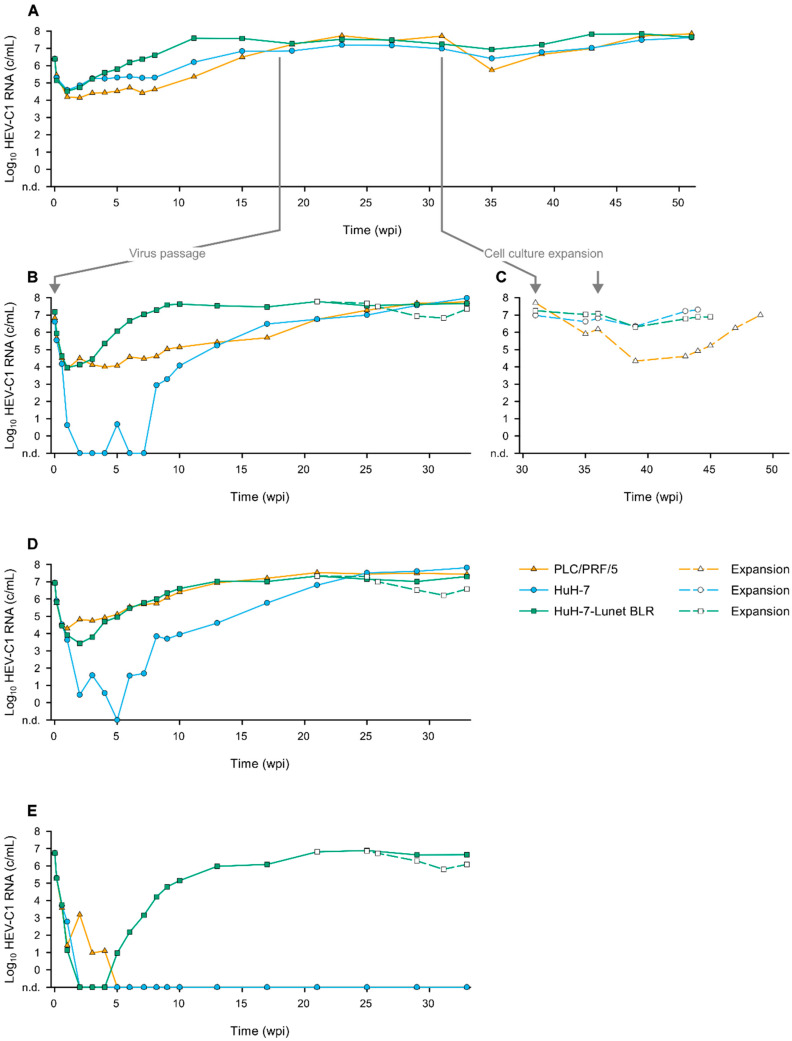
Replication dynamics of the rat HEV (HEV-C1) in different human liver carcinoma cell lines. (**A**) PLC/PRF/5, HuH-7, and HuH-7-Lunet BLR were inoculated with the PLC/PRF/5 cell culture supernatant positive for HEV-C1 strain R63. Supernatants of all three cell lines were collected on day 126 post inoculation and used to (**B**) passage the virus in a cell line-specific manner, meaning that the HEV-C1 strain R63 positive PLC/PRF/5, HuH-7, and HuH-7-Lunet BLR cell culture supernatants were inoculated onto PLC/PRF/5, HuH-7, and HuH-7-Lunet BLR, respectively. (**C**) The HEV-C1 strain R63 positive cell cultures from the first inoculation were expanded from T12.5 to T75 flasks and a second time from T75 to T175 flasks. As soon as the initial viral load was reached again in the supernatant, the cells were detached and aliquots stored at −192 °C. (**D**) R63 passage 6 (derived from PLC/PRF/5) and (**E**) R68 positive liver homogenate were also inoculated onto PLC/PRF/5, HuH-7, and HuH-7-Lunet BLR.

We selected the following cell lines with a realistic chance of successful rat HEV replication based on preexisting reports: PLC/PRF/5 and HuH-7 [5,15,16]. Moreover, we introduced HuH-7-Lunet BLR as a new candidate cell line in this context, since it is highly permissive to HEV [19]. Cells were inoculated with HEV-C1 strain R63 passaged once (p1) in PLC/PRF/5. Successful replication was observed in all three cell lines including the newly introduced HuH-7-Lunet BLR cells (Figure 1A). Intriguingly, the virus replicated fastest in HuH-7-Lunet BLR to a maximum viral load of 4 × 10^7^ c/mL within 11 weeks post inoculation (wpi). Interestingly, between three and eight wpi, R63 p1 replicated at a constant lower level of around 2 × 10^5^ and 3 × 10^4^ c/mL in HuH-7 and PLC/PRF/5, respectively. However, after eight wpi, the viral replication rates in HuH-7 and PLC/PRF/5 gained momentum and reached similar maximum viral loads as in HuH-7-Lunet BLR after 23 wpi. It took approximately twice as long to reach this plateau compared to HuH-7-Lunet BLR. After the expansion of the cells, we observed a temporary drop in the viral loads, which was most prominent in PLC/PRF/5 (Figure 1C). The maximum viral load was reached again after around 20 weeks.

Strain R63 p2 was passaged in a cell-specific manner (i.e., HuH-7-derived R63 was inoculated onto HuH-7 and so forth) (Figure 1B). After passaging, the interesting initial observation of the fastest replication in the HuH-7-Lunet BLR cells was confirmed. Comparable to the initial experiment (Figure 1A), the maximum viral load after passaging was reached at 4 × 10^7^ c/mL after nine weeks. It took considerably more time to generate similarly high viral loads in HuH-7 and PLC/PRF/5: the maximum was reached after 25–30 weeks, which was approximately thrice as long as compared to HuH-7-Lunet BLR. This experiment again showed that R63 replicated at a lower level in PLC/PRF/5, this time until around six wpi. The viral RNA concentration was mostly below the lower limit of the PCR detection in the HuH-7 supernatant during this period.

To further investigate whether R63 replicates fastest to maximum viral loads in HuH-7-Lunet BLR, the cells were inoculated with PLC/PRF/5-adapted strain R63 p6 (passaged six times in PLC/PRF/5; kindly provided by Prof. Dr. Reimar Johne). Interestingly, the replication dynamics in HuH-7-Lunet BLR and PLC/PRF/5 were comparable in this experiment (Figure 1D). While a maximum of 1 × 10^7^ c/mL was generated by HuH-7-Lunet BLR at 13 wpi, the viral load further increased in PLC/PRF/5 and reached a maximum of 3 × 10^7^ c/mL at 21 wpi. HuH-7 reached a plateau at 25 wpi and further slightly rose until the end of the observation period to 6 × 10^7^ c/mL at 33 wpi. This showed that HuH-7-Lunet BLR equally supports the replication of the PLC/PRF/5-adapted R63 p6 strain.

Finally, we evaluated the cell lines for the susceptibility to de novo isolation of the HEV-C1 strain R68 from the rat liver tissue. HuH-7-Lunet BLR was the only cell line to support a robust viral replication, reaching a maximum of around 8 × 10^6^ c/mL at 25 wpi (Figure 1E). Isolation of R68 in HuH-7 and PLC/PRF/5 was not successful. At least, PLC/PRF/5 weakly supported the viral replication in the first weeks post inoculation but dropped below the limit of detection of five wpi and never recovered over the whole observation period.

## 4. Discussion

In this study, we asked whether our PLC/PRF/5-based cell culture system optimized for HEV replication [19]—compared to HuH-7 and HuH-7-Lunet BLR—was (i) able to propagate cell culture-derived rat HEV, and (ii) if it was susceptible to de novo isolation of the virus from rat liver tissue. Our results show that (i) PLC/PRF/5-derived rat HEV could be passaged in the optimized HEV cell culture system in all three cell lines, but (ii) only the cell clone HuH-7-Lunet BLR was susceptible to the de novo isolation of rat HEV from the rat liver homogenate.

Our first finding that PLC/PRF/5-derived rat HEV can be passaged in the optimized HEV cell culture system in all three cell lines is supported by several experiments including a virus passage and cell culture expansion. We demonstrate for the first time that rat HEV can be successfully propagated in HuH-7-Lunet BLR cells. Indeed, the inoculation of rat HEV strain R63 p1 (once passaged in PLC/PRF/5) onto PLC/PRF/5, HuH-,7 and HuH-7-Lunet BLR identified HuH-7-Lunet BLR as the cell line reaching the highest viral loads of approximately 3 × 10^7^ c/mL in the shortest period of time, followed by HuH-7. Surprisingly, the least efficient replication was observed in PLC/PRF/5. These findings differ from those reported by Jirintai et al., who observed a similar replication pattern of rat HEV in PLC/PRF/5 and HuH-7 with an inoculum passaged once in PLC/PRF/5 [15]. However, we confirmed our findings by a further cell line-specific passage of R63. Only R63 p6 (adapted to PLC/PRF/5 over six passages) replicated with comparable efficiency in PLC/PRF/5 and HuH-7-Lunet BLR. Another aspect in favor of HuH-7-Lunet BLR (and HuH-7) over PLC/PRF/5 is the observation that the expansion of rat HEV positive PLC/PRF/5 led to a sharp decrease in viral load, which was not observed in HuH-7 and HuH-7-Lunet BLR.

Our second finding that only cell clone HuH-7-Lunet BLR was susceptible to de novo isolation of HEV-C1 from the rat liver homogenate stands in contrast to the hitherto published attempts. We only identified three other studies that aimed at isolating HEV-C1 from rat specimens. First, isolation was attempted from rat feces, but was unsuccessful in three different rat liver cell lines: N1-S1 (ATCC CRL-1604), clone 9 (ATCC CRL-1439), and MH1C1 (ATCC CCL-144) [14]. Second, Jirintai et al. successfully isolated rat HEV from rat liver tissue in PLC/PRF/5 [15], while, third, this finding could not be reproduced by Debing et al. [16]. However, the latter group reported the isolation of rat HEV from the rat liver tissue in HuH-7 and—to a lesser extent—in HepG2/C3A. The virus could not be propagated in a rat hepatoma cell line (H4IIE). Our findings are partly in line with the results reported by Debing et al. and Jirintai et al. For example, we were also able to successfully passage PLC/PRF/5-derived rat HEV in various cell lines. However, in our study, de novo isolation was only possible in HuH-7-Lunet BLR. An exemption to these isolation attempts was the isolation of HEV-C1 from human feces in A549, HuH-7, and Caco-2 [5], since rat-derived HEV-C1 could not be propagated in A549 [15]. The authors speculate that the patient’s immunosuppression possibly enabled the virus to surmount the species barrier.

The overall higher susceptibility of HuH-7-Lunet BLR to rat HEV is certainly dependent on several virus and host cell factors. One part could be the expression of higher levels of liver-specific microRNA-122 (miR-122) compared to the parental HuH-7 and PLC/PRF/5 [22]. Hepatitis C virus (HCV) replication depends on the presence of miR-122 [23] and miR-122 also enhances the replication of HEV genotypes 1 and 3 [24]. Rat HEV genomes could also harbor the miR-122 target site and profit from higher miR-122 expression levels. Another factor could be that quasi-enveloped rat HEV derived from the cell culture supernatant may enter into a broader range of cells via endocytosis while the naked rat HEV derived from the rat liver homogenate may be dependent on the cell surface molecules, which were only sufficiently present on the HuH-7-Lunet BLR compared to the parental HuH-7 and PLC/PRF/5. On the other hand, this could also be a virus strain-specific issue since rat HEV strains R63 and R68 differ in 37/1636 (2.26%), 7/644 (1.09%), and 6/102 (5.88%) positions on the amino acid level in open reading frames 1 (non-structural proteins), 2 (capsid), and 3 (multifunctional phosphoprotein), respectively. Further studies are needed to examine these hypotheses.

One limitation of this study was that we were very limited in the non-cell culture-derived rat HEV positive material. This enabled us to perform only one de novo isolation of the rat HEV from rat liver tissue.

In summary, our study shows the propagation of rat HEV in three cell lines. PLC/PRF/5 and HuH-7 were only susceptible to cell culture-derived rat HEV. We identified HuH-7-Lunet BLR as the most permissive cell line for rat HEV. De novo isolation of the virus from the rat liver was only successful with this cell line. Our HEV-C1 cell culture system may be useful for basic research, the generation of animal-independent large virus stocks as well as for the testing of antiviral compounds and drugs.

## Data Availability

Data is available upon request.
